# Synchrotron-based operando X-ray diffraction and X-ray absorption spectroscopy study of LiCo_0.5_Fe_0.5_PO_4_ mixed d-metal olivine cathode

**DOI:** 10.1038/s41598-023-28951-z

**Published:** 2023-02-07

**Authors:** Taymour A. Hamdalla, Abdelaziz M. Aboraia, V. V. Shapovalov, A. A. Guda, N. V. Kosova, O. A. Podgornova, A. A. A. Darwish, S. A. Al-Ghamdi, S. Alfadhli, Aadel M. Alatawi, Alexander Soldatov

**Affiliations:** 1 Present Address: Department of Physics, Faculty of Science, University of Tabuk, 71491 Tabuk, Saudi Arabia; 2 Department of Physics, Faculty of Science, Alexandria University, Alexandria, Egypt; 3 Physics Department, Faculty of Science, Al-Azhar University, Assiut, 71542 Egypt; 4 The Smart Materials Research Institute, Southern Federal University, Sladkova, Russia; 5 Institute of Solid State Chemistry and Mechanochemistry, Siberian Branch of the Russian Academy of Sciences, 18 Kutateladze, 630128 Novosibirsk, Russia; 6 Department of Physics, Sana’a University, Sana’a, Yemen; 7 Electrical Engineering Department, Faculty of Engineering, Tabuk University, Tabuk, Saudi Arabia

**Keywords:** Energy science and technology, Materials science, Physics

## Abstract

Lithium-ion batteries based on high-voltage cathode materials, such as LiCoPO_4_, despite being promising in terms of specific power, still suffer from poor cycle life due to the lower stability of common non-aqueous electrolytes at higher voltages. One way to overcome this issue might be decreasing the working potential of the battery by doping LiCoPO_4_ by Fe, thus reducing electrolyte degradation upon cycling. However, such modification requires a deep understanding of the structural behavior of cathode material upon lithiation/delithiation. Here we used a combination of *operando* synchrotron-based XRD and XAS to investigate the dynamics of d-metal local atomic structure and charge state upon cycling of LiCo_0.5_Fe_0.5_PO_4_ mixed d-metal olivine cathode material. Principal components analysis (PCA) of XAS data allowed the extraction of spectra of individual phases in the material and their concentrations. For both Co and Fe two components were extracted, they correspond to fully lithiated and delithiated phases of Li_x_MPO_4_ (where M = Fe, Co). Thus, we were able to track the phase transitions in the material upon charge and discharge and quantitatively analyze the M^2+^/M^3+^ electrochemical conversion rate for both Fe and Co. Rietveld's refinement of XRD data allowed us to analyze the changes in the lattice of cathode material and their reversibility upon (de)lithiation during cycling. The calculation of DFT and Bader charge analysis expects the oxygen redox procedure combined with d-metals redox, which supplements iron charge variations and dominates at high voltages when x < 0.75 in Li_x_CoFePO_4_.

## Introduction

The improvement of Li-ion batteries (LIB) is focused on enhancing the specific power and energy density. To improve the battery energy density, it's necessary to utilize a high voltage positive electrode material. LiCoPO_4_ (lithium cobalt phosphate, LCP) is of particular interest due to a high theoretical capacity of around 167 mAh g^−1^, and high operating voltage (OV) ~ 4.8 V vs. Li+/Li^1–6^. LiCoPO_4_ belongs to the LiMPO_4_ olivine family (M = Co, Mn, Fe, and Ni) and demonstrates mediocre rate performance and cycle stability because of low diffusion of lithium ions in the lattice and poor electronic conductivity^7–9^. To increase the electrochemical performance of LiCoPO_4_, different strategies were used, such as coating of LCP with various carbon sources to increase electronic conductivity^1,2,10–14^ and nanosizing to improve ionic conductivity.

Moreover, in several works, cobalt ions were substituted with different transition metals such as Ni, Mn, and Fe, and this substitution caused significant improvement in the electronic and ionic conductivity of LiCoPO_4_, thus enhancing its electrochemical performance^15–19^. The improvement in the bulk conductivity is not due to antisite defects (Co or Fe on the Li site); but from the mobile polarons, and associated with lithium vacancies^20,21^. While the operating voltage was not noticeably lowered, the charge carrier transport in the crystal structure was improved, leading to a stabilization of the charging process and a decrease in the number of reactive species formed on the electrode surface.

During the electrochemical reactions, the electrode materials suffer from a significant structural change. In order to reliably track this structural evolution and relate it to the specific chemical transitions, it is necessary to use the operando approach. Operando XRD allows one to study full dynamics of the sample structure, track intermediate phases and avoid sample relaxation. This allows a deeper understanding of the internal mechanisms affecting the electrochemical characteristics of the material and its degradation, providing an understanding of the improvement of the properties of the material.

The changes in the crystallography that occur upon the LiCoPO_4_ to CoPO_4_ transition have been studied extensively^6,14,22,23^. Bramink and co-authors found that pure LiCoPO_4_ undergoes an electrochemical de-lithiation process by two subsequent two-phase steps, creating a Li_2/3_CoPO_4_ intermediate structure^24^. Strobridge and co-authors^25^ detected two intermediate phases in Fe-substituted LiCoPO_4_ upon de-lithiation. The 1st phase is Li_2/3_Fe_x_Co_1−x_PO_4_ for all concentrations, but the 2nd phase is Li_1−x_(Fe^3+^)_x_(Co^2+^)_1−x_PO_4_, for 0 < x < 1. Moreover, it was observed that for low lithium concentrations (0 < x < 0.5), de-lithiation between Li_1−x_(Fe^3+^)_x_(Co^2+^)_1−x_PO_4_ and Li_2/3_Fe_x_Co_1−x_PO_4_ occurs through a bulk single-phase mechanism^25^. Kang et al. have demonstrated by theoretical and experimental techniques that the doping by Fe^2+^ in the olivine structure reduces the Li-Co antisite mixing^26^. This effect refers to the preferred occupation of the cobalt site by iron, which leads to the expansion of an oxygen octahedron and improves the diffusion of Li^+^ in channels^27^. Furthermore, Allen and co-authors observed that Fe^3+^ has the ability to inhibit capacity fading, and the LiCoPO_4_ doped by Fe has better electronic conductivity than the pure LiCoPO_4_^28^.

An in-depth understanding of the relationship among the structural characteristics and electrochemical routine is crucial for the rational and conscious development of doped LCPO materials; therefore, a combination of operando X-ray diffraction and absorption spectroscopy is useful to study the dynamics of the local atomic and electronic structure of electrode material during electrochemical transformations. In this paper, we employ such a combination at the synchrotron source to investigate the Fe doped LCP cathode material with a general formula LiFe_0.5_Co_0.5_PO_4_. We also use ab initio DFT calculations to address the peculiarities of redox processes in this dual-ion system.

## Materials and methods

### LCFP synthesis

The LiCo_0.5_Fe_0.5_PO_4_ was synthesized by mechanochemically assisted carbothermal reduction. The reagents of Li_2_CO_3_, Fe_2_O_3_ (a-modification), Co_3_O_4_, and (NH_4_)_2_HPO_4_ (qualification “pure for analysis”) were mixed with carbon “P 277” by a high-energy AGO-2 planetary mill under argon atmosphere for 5 min in a molar ratio (Co + Fe):P:Li = 1:1:3. The resulting product was annealed under argon flow for one hour at 750 °C. The final product was a single-phase LiCoFePO_4_ with an orthorhombic structure^10^.

### Characterization using electrochemistry

Electrodes for the electrochemical characterization were made through mixing around 80 wt% of LCFP active component with 15 wt% of carbon (Timcal Super P Conductive) and 5 wt% of PVDF/NMP binder. The resulting slurry was cast on the aluminum foil and dried at 150 °C under a vacuum overnight. The sample mass loading was around 2–3 mg per cm^2^ after drying, and an electrode with a diameter of 10 mm was employed. The cells were assembled in an argon glovebox with a lithium foil as an anode, a glass fiber filter (Whatman, Grade GF/C) as a separator, and 10 μl of 1 M LiPF_6_ solution in 1:1 (by weight) EC:DMC (Sigma Aldrich) as an electrolyte. The assembled cells were cycled in a galvanostatic mode from 3.0 to 5.0 V vs. Li/Li^+^ at RT with a charge/discharge rate of C/10.

### Operando synchrotron-based XAS and XRD characterization

X-ray absorption spectra and simultaneous diffraction patterns were obtained in the operando mode at the European Synchrotron Radiation Facility ESRF's Swiss-Norwegian Beamline BM31 (Grenoble, France). Home made electrochemical cells with X-ray transparent windows made from glassy carbon were used for cycling^29^. The resulting cathode powder was placed in the cell with a mass load of 13 mg/cm^2^. The cells were cycled galvanostatically using a BAT-SMALL battery analyzer (Astrol Electronic AG, Switzerland) in the region from 3 to 5 V vs Li/Li^+^ with a cycling rate of C/10. Diffraction patterns were recorded each 5.5 min, while the Fe and Co K-edge absorption spectra—for 9.5 min, resulting in a total measurement time of 15 min per point.

2D diffraction images were performed with 5 s acquisition time by the Dexela 2D CMOS detector (PerkinElmer, USA). The wavelength λ = 0.49796 Å and the sample-to-detector distance of 289.55 mm were calibrated using silicon powder and LaB_6_ standards.

Fe and Co K-edge X-ray absorption spectra were measured in a single continuous scan in transmission mode employing a Si(111) monochromator. Ionization chambers were used to monitor intensity before and after assembling, and a Gd_2_O_3_ reference was measured simultaneously with the sample for energy alignment.The observed spectra were aligned, flattened, and normalized using the Demeter package's Athena function^30–32^. Then all spectra were interpolated on the same set of energy points and submitted as the input for the principle component analysis (PCA) via open-source PyFitIt software as described precisely in the Mathematical details of the PCA procedure are presented below, and were not included in the main text, but were appropriately referenced:

Any XANES spectrum can be projected onto an orthogonal basis set of functions. The set of projections over each basis function then describes the entire spectrum. We construct basis set functions in the procedure of principal component analysis applied to the theoretical training set. A theoretical XANES dataset $\mathbf{X}$ is decomposed in the following way:

1 $$\mathbf{X}={\mathbf{U}}_{(\mathrm{m}\times \mathrm{m})}{{\varvec{\Sigma}}}_{(\mathrm{m}\times \mathrm{n})}{{\mathbf{V}}^{\mathrm{T}}}_{(\mathrm{n}\times \mathrm{n})}$$

where $\mathbf{X}$ can be considered as a matrix of dimensions $(m\times n)$, where *m* is the number of XANES energy points while *n* is the number of theoretical spectra constituting it, $\mathbf{U}$ and $\mathbf{V}$ are two square unitary matrices, ${\varvec{\Sigma}}$ is a diagonal rectangular matrix while $\mathrm{T}$ denotes the transpose operator. The diagonal elements of ${\varvec{\Sigma}}$ are referred to as the singular dataset values, whose magnitudes are proportional to the amount of variance of the related component. Columns in matrix $\mathbf{U}$ have the dimensionality of a XANES spectrum and are referred to as abstract mathematical components (i.e. they do not look like line spectra, but their proper linear combination does). Matrix ${\varvec{\Sigma}}\mathbf{V}$ provides the weights which need to be employed to reconstruct each spectrum of $\mathbf{X}$ from $\mathbf{U}$. Considering Eq. (1), the *i*th XANES spectrum ${{\varvec{\upmu}}}_{\mathrm{i}}$ of $\mathbf{X}$, can be rewritten as^31^:

2 $${{\varvec{\upmu}}}_{\mathrm{i}}\left(\mathbf{E},\mathbf{p}\right)=\sum_{\mathrm{j}=1}^{\mathrm{m}}{\mathrm{h}}_{\mathrm{ij}}(\mathbf{p}){\mathbf{u}}_{\mathrm{j}}(\mathbf{E})$$

where $\mathbf{E}=({\mathrm{E}}_{1},\dots {\mathrm{E}}_{\mathrm{m}})$ is the set of XANES energy points, ${\mathbf{u}}_{\mathrm{j}}$ represents the jth column vector of $\mathbf{U}$ while ${h}_{\mathrm{ij}}$ is the fraction of the *j*th component in the *i*th spectrum provided by matrix ${\varvec{\Sigma}}\mathbf{V}$^32^.

### Ab initio DFT calculations

The VASP software was used to perform ab initio DFT calculations^33,34^ using GGA-PBE projector-augmented wave pseudopotentials with spin polarization. GGA + U correction was 5.05 and 3.84 for Co and Fe, respectively^6^. A two-step geometry optimization incorporating cell volume and then atomic locations was done for each alkali metal concentration. For ionic relaxation and electronic minimization, the conjugate gradient method and the specific Davidson block iteration scheme techniques were utilized. Following that, optimized structures were employed for precise single-point computations of total energy, charge densities, and atomic charges using the Bader technique^35,36^. To determine Bader charge values for reference compounds including Fe and Co with known oxidation states the same computational techniques were used.

## Result and discussion

**Figure 1:**
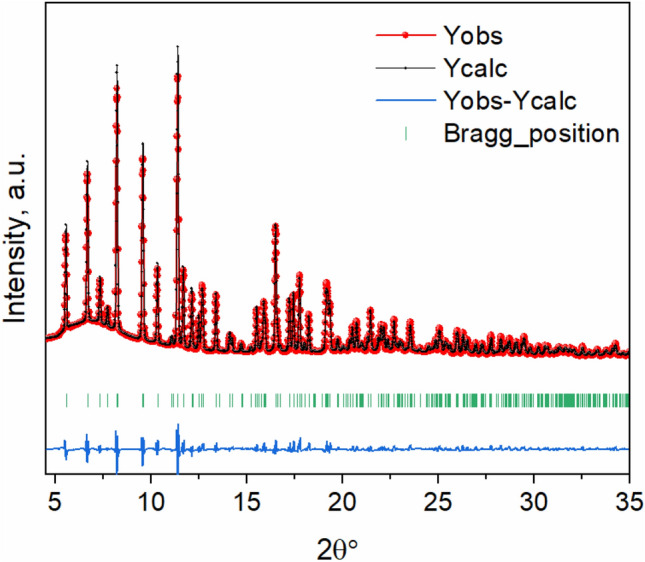
Rietveld refinement of the synchrotron XRD pattern of as-synthesized LiCo_0.5_Fe_0.5_PO_4_.

According to synchrotron powder XRD (Fig. 1), the as-synthesized LiFe_0.5_Co_0.5_PO_4_ is characterized as a single orthorhombic phase without any carbon peaks according to the open crystallography database reference #9004884. Rietveld refinement of the XRD profile suggests that it has a P*bnm* space group with the lattice parameters of *a* = 4.69 Å, *b* = 10.25 Å, and *c* = 5.96 Å.

**Figure 2:**
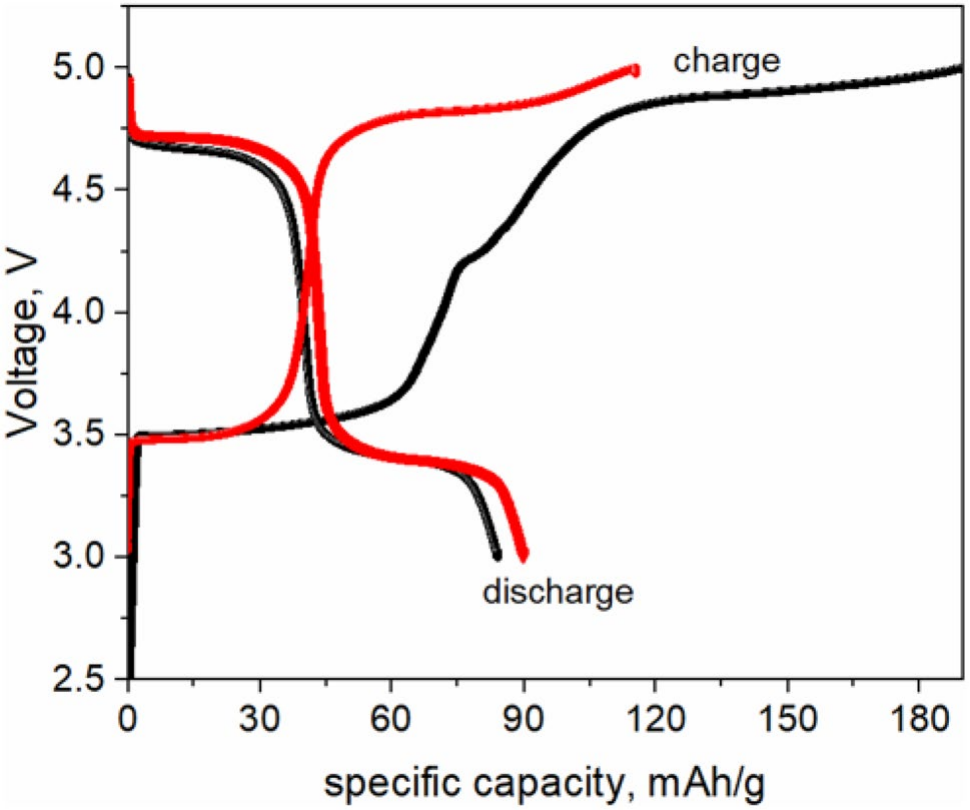
First two charge/discharge profiles of LiCo_0.5_Fe_0.5_PO_4_.

Figure 2 displays the first two charges/discharge profiles of LiCo_0.5_Fe_0.5_PO_4_ cathode. Two voltage plateaus were observed, the first one at ~ 3.5 V corresponds to the Fe^2+^/Fe^3+^ redox couple, and the second one at ~ 4.9 V—to the Co^2+^/Co^3+^ redox couple. This material demonstrates the capacity of around 89 mAh/g and 93 mAh/g for the first and the second discharges, respectively. The 1st specific charge capacity is slightly higher than the theoretical capacity of 167 mAh/g, which indicates that some side reactions occur, which might include the CEI (cathode EI) formation and electrolyte decomposition at high voltage. However, in the second cycle, the charge-specific capacity decreases.

To reveal the structural transformations upon lithiation/delithiation, we carried out operando synchrotron diffraction experiment. As reported in the previous work, ex situ powder XRD data for several cobalt phosphate compositions (LiCoPO_4_, Li_0.7_CoPO_4_, and CoPO_4_) exhibit reflections from two olivine phases with various unit cell volume and a substantial contribution from the fraction of an amorphous phase^37^. We assume that the cathode undergoes partial amorphization during lithium extraction.

### Operando X-ray diffraction

**Figure 3:**
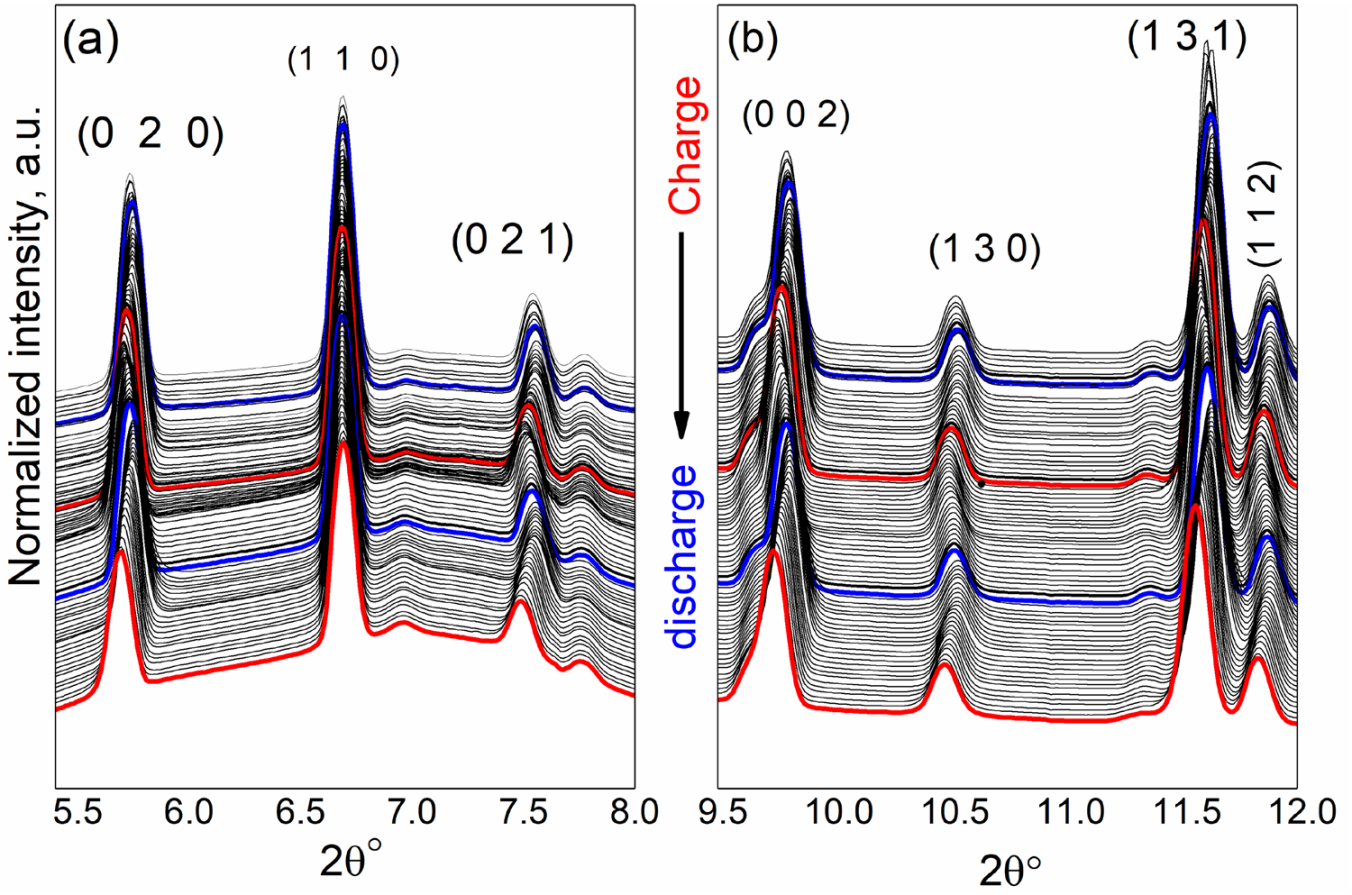
Operando synchrotron XRD patterns of LiCo_0.5_Fe_0.5_PO_4_ upon the first two charge–discharge cycles in the 2θ range: (**a**) from 5.3 to 8°, indicating the shift to the lower 2θ values at this region, (**b**) from 9.5 to 12° indicating the shift to lower 2θ values at this region. The red line represents the charging process, and the blue line is the discharge process.

**Figure 4:**
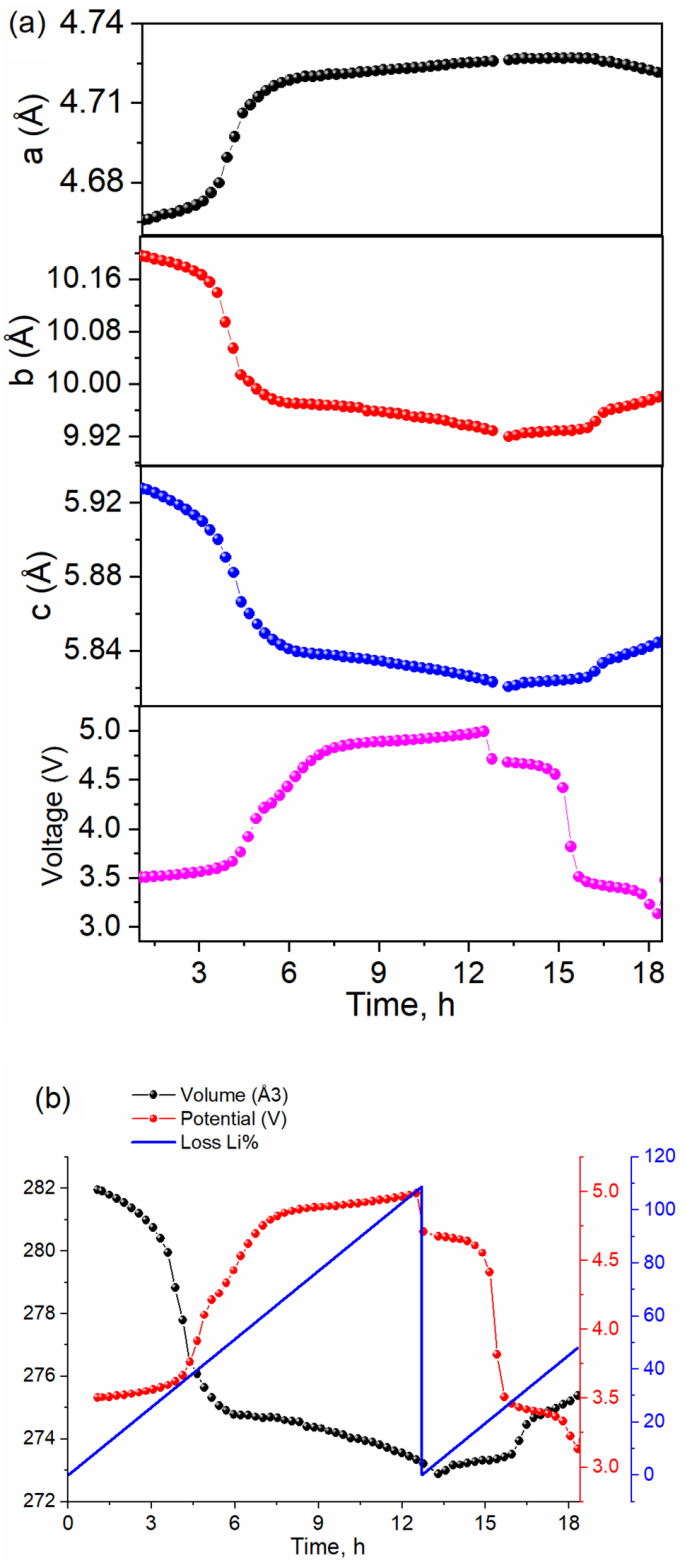
Evolution of (**a**) lattice parameters and (**b**) unit cell volume obtained from the operando XRD compared to the cell potential and Li loss upon the cycling of LiCo_0.5_Fe_0.5_PO_4_.

Figure 3 displays the evolution of the selected reflections in the operando synchrotron XRD patterns during the first two lithiation/de-lithiation cycles of a cell at the C/10 rate. No amorphization of the active material was observed. At several planes (“0 2 0”, “0 2 1”, and “1 3 0”), this observation is in agreement with Strobridge et al.^25^. During the first charge, around 0.5 Li per formula unit leaves the structure of LiCo_0.5_Fe_0.5_PO_4,_ accompanied by an increase in the lattice parameter “a” to 4.72 Å and a decrease of b and c to 9.92 Å and 5.82 Å, respectively, as shown in Fig. 4a. Since the lithium loss was approximately 0.3 per formula unit in the first discharge, this phase partially recovered to the original LiCo_0.5_Fe_0.5_PO_4_ state, with the following lattice parameters a = 4.715 Å, b = 10.005 Å, and c = 5.855 Å These results are similar to those previously reported for *Pnma*-Li_0.7_CoPO_4_ structure with a = 10.070 Å, b = 5.851 Å and c = 4.717 Å^38^. It is clear that upon charging, the lattice parameter a is expanding, while b and c are shrinking. At the end of charge, the b and c cell parameters were higher than expected for Fe_0.5_Co_0.5_PO_4_, but c was smaller, revealing that the full conversion was not achieved during the operando experiment. Moreover, upon lithiation and delithation processes, the profiles of the potential remain stable, which proposed small or insignificant changes in the mechanism of the reaction, as demonstrated in Fig. 4b.

### In situ synchrotron X-ray absorption measurements

**Figure 5:**
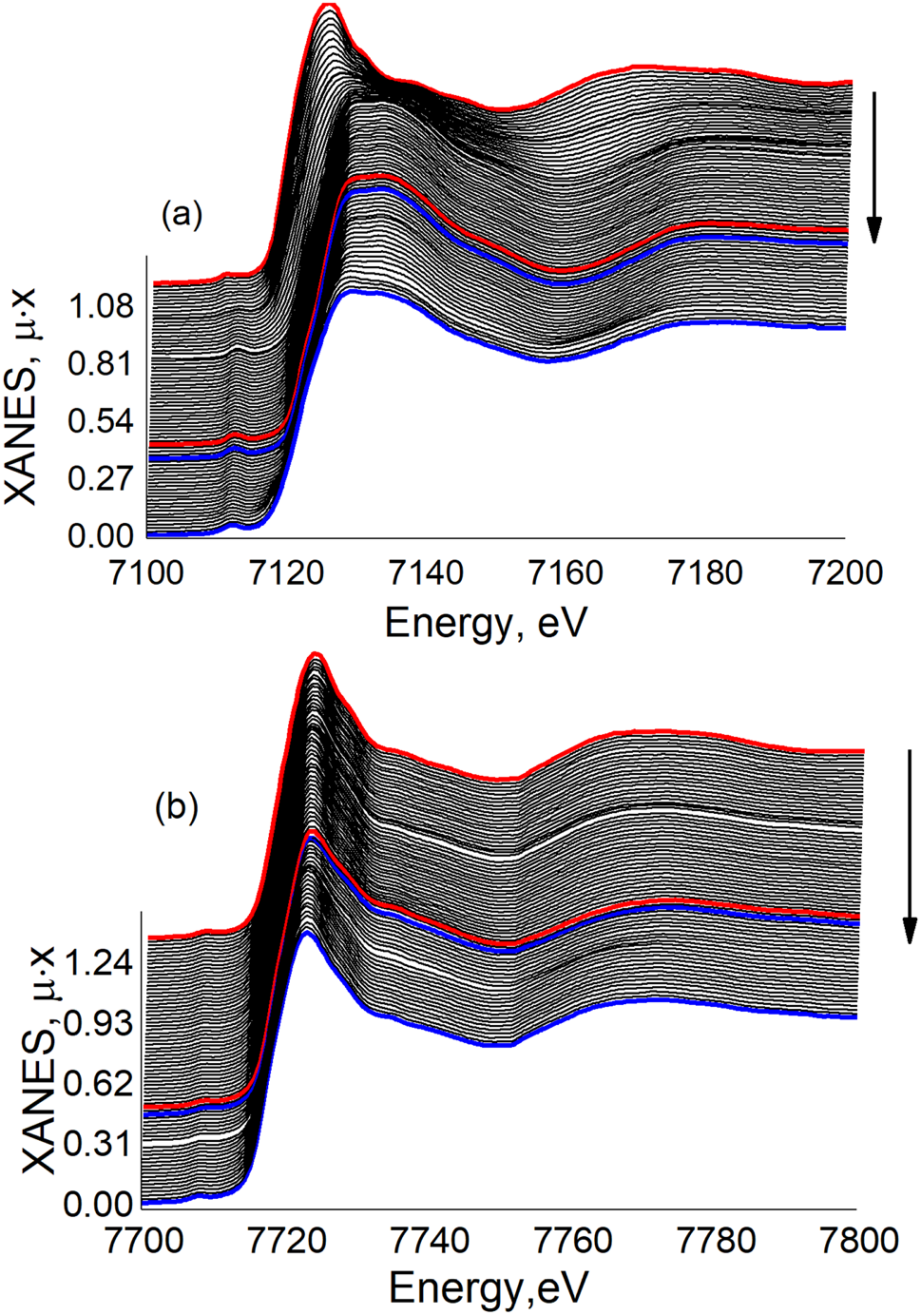
A series of operando XANES spectra for the Fe (**a**) and Co (**b**) K-edge in the LiCo_0.5_Fe_0.5_PO_4_ cathode material obtained during the first cycle (the red line is a charging process, and the blue line represents the discharge process).

Figure 5 demonstrates the evolution of operando Fe and Co K-edge XANES spectra of LiCo_0.5_Fe_0.5_PO_4_ cathode upon cycling. Though for Fe, there is a very notable chemical shift of the absorption edge, indicating the Fe^2+^/Fe^3+^ redox reaction (Fig. 5a), for Co K-edge, this shift is hardly noticeable (Fig. 5b).

**Figure 6:**
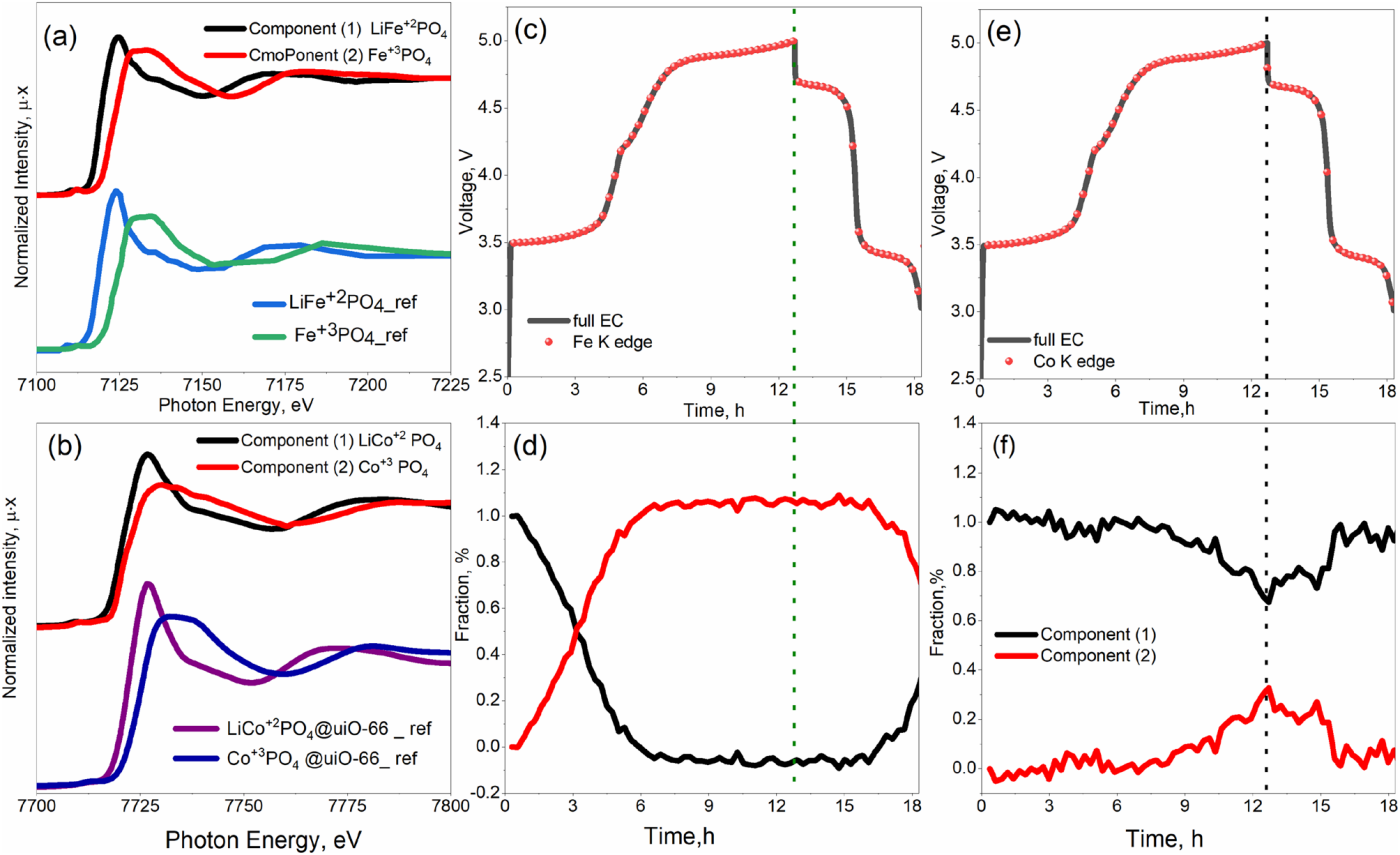
PCA components extracted from the series of operando XANES spectra for the Fe compared to the experimental spectra for LiFePO_4_ and FePO_4_ (ref.^40^) (**a**) and Co (**b**) K-edge in the LiCo_0.5_Fe_0.5_PO_4_ cathode material, compared to the experimental spectra of the reference compounds (LiCoPO_4_@UiO-66)^7^. (**c**) Cell voltage of Fe during the first cycle dots on a black voltage profile mark the start time for measuring each successive XAS spectrum. (**d**) PCA phase concentration of the Fe^2+^ and Fe^3+^ components compared to the cell potential. (**e**) Cell Voltage of Co during the first cycle dots on a black voltage profile marks the start time for measuring each successive XAS spectrum. (**f**) PCA phase concentration of the Co^2+^ and Co^3+^ components compared to the cell potential.

This set of spectra was further processed using PCA. Because the XAS spectrum of a multiphase bulk mixture is a superposition of XAS spectra for each phase, PCA can be used to mathematically decompose the sequence of spectra for the electrochemical phase transition process to get the pristine spectra of the relevant phases and their concentrations. Initially, all spectra were interpolated to a single energy range. Singular value decomposition was then used to obtain a certain number of components from the sequence of spectra. In our situation, two abstract spectra were more transformed into chemically meaningful information via target transformation. The following physical constraints were expected: spectra should be normalized, the values of concentrations should be positive, and at least one of the components under consideration should represent either a fully reduced or fully oxidized phase. In this way, two components were successfully identified for both Co and Fe, as shown in Fig. 6.

There is a significant change between the first and the second components because the first component corresponds to Fe^2+^ and the second—to Fe^3+^ in CoFePO_4_, as demonstrated by the comparison with the experimental XAS spectra for LiFePO_4_ and FePO_4_ in Fig. 6a. For the Co K-edge, in a similar way, the first component can be attributed to the Co^2+^ in LiCo_0.5_Fe_0.5_PO_4_ and the second one—to the Co^3+^ in CoFePO_4_ due to a rare study of in situ XAS of LiCoPO_4_, the current result compared to our previously reported work LiCoPO_4_ coating by UiO-66 meatlorganic framework^7^, as shown in Fig. 6b.

Figure 6d shows that at the first charge, the Fe^2+^ ions are almost completely converted to Fe^3+^, thus, about 50% of Li ions should be extracted from the cathode material. Figure 6e,f demonstrate that the electrode surface remains predominately Co^2+^ in LiCoPO_4_, irrespective of the charge process. However, upon discharge process has noted a shift in the center of peaks to the higher energy suggesting the growth of some Co^3+^, with additional loss of various characteristics in the bulk of the material. The determination of several features missing in Co^3+^ compounds suggests that (~ 60)Co^2+^ was not fully oxidized^39^. There is also a peculiar disturbance in Co phase concentration in the region corresponding to the Fe^2+^ redox activity. Such a reaction of Co on the change in the local atomic and electronic coordination of Fe might be indicative of the homogeneous distribution of d-metals in the structure of cathode material and the homogeneous Li insertion/extraction mechanism. X-ray diffraction is sensitive to the long-range order, and the conclusion about the solid solution mechanism is made based on this experimental technique. On the contrary, X-ray absorption fine structure is sensitive to the local atomic structure within 5–7 Å around the absorber. Therefore the “components” retrieved from PCA or linear combination fits are related to the local order around 3d metals but not to crystalline phases.

**Figure 7:**
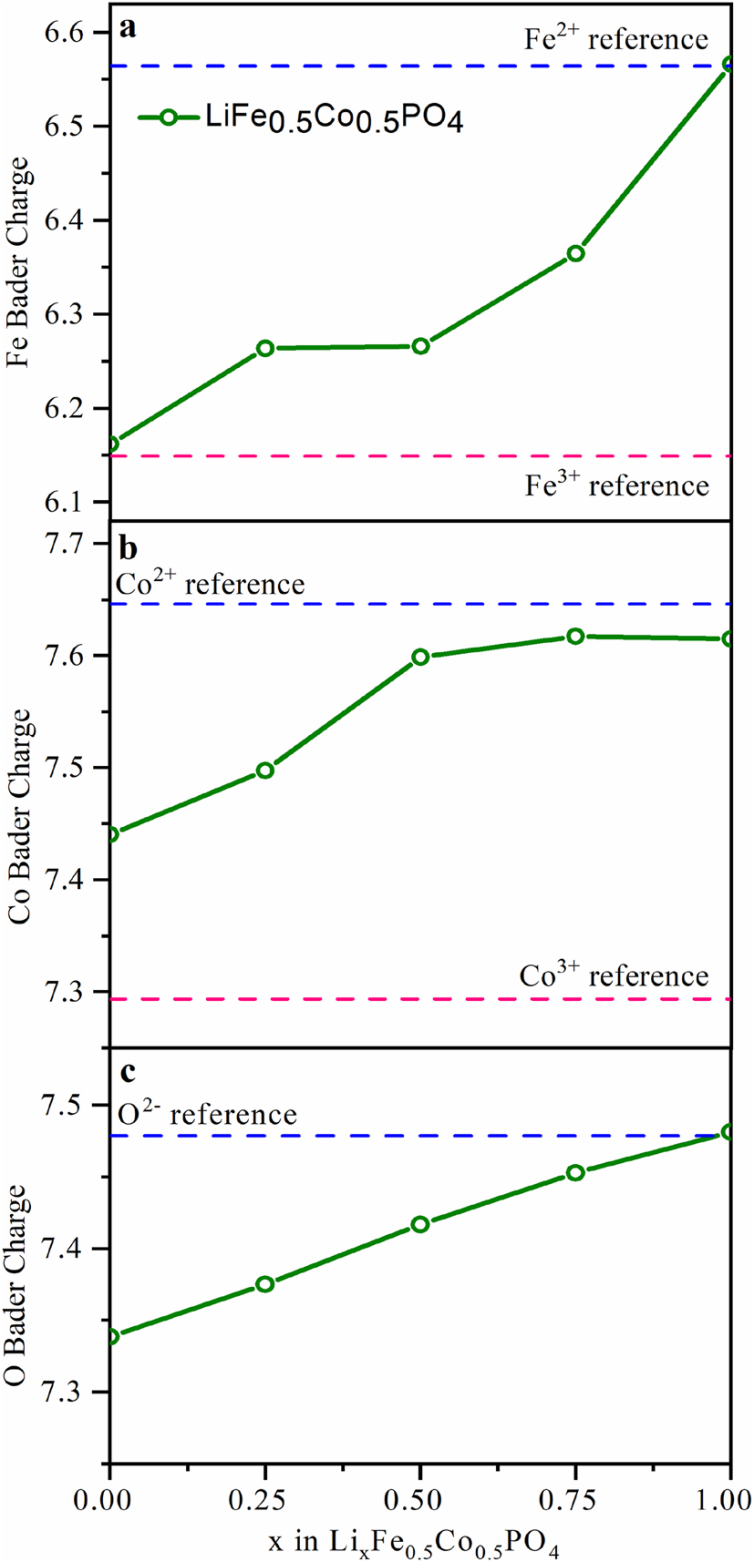
Unit cell averaged local atomic (Bader) charges for Fe (**a**), Co (**b**) and O (**c**) as a function of x in Li_x_Co_0.5_Fe_0.5_PO_4_ (x = 0, 0.25, 0.5, 0.75, and 1). The values for each species were averaged over non-equivalent atomic positions. Reference charge values (dashed lines) were obtained from calculations for reference compounds with different Co and Fe oxidation states.

To understand both cobalt and iron performance during the charge/discharge process of LiCoFePO_4_, ab initio DFT calculations were carried out to estimate the local atomic charges according to the Bader method for different Li stoichiometry in Li_x_CoFePO_4_ (x = 0, 0.25, 0.5, 0.75 and 1). Figure 7 demonstrates the unit cell averaged atomic charges for cobalt, iron, and oxygen compared to the reference values. The calculation suggests that upon the lithiation, Fe^2+^ should fully convert to Fe^3+^, which is in agreement with the PCA results shown above. However, the suggested Co^3+^/Co^2+^ conversion rate during the lithiation is only slightly above 50%, similar to what was observed in the operando XAS measurements. However, for the oxygen, there is also a substantial change in the Bader charge upon the delitiation, which suggest that there is a reversible anion redox reaction on oxygen, which takes part in the total charge compensation mechanism to make up for the deficiency in Co oxidation.

## Conclusion

The current work reports *operando* characterization of lithium cobalt iron phosphate charge/discharge process as a positive electrode in Li-ion half-cell by synchrotron diffraction and absorption in terms of Co and Fe k-edge. 115 patterns and 200 spectra of synchrotron diffraction and absorption obtained over 36 h of electrochemical cycling exhibited the reaction of reversible changes in redox, structural, and electronic properties for Co and Fe sites during the first cycle. The electrochemical performance was tested. Cycling curves exhibited two voltage plateaus, one corresponding to the Fe^2+^/Fe^3+^ redox at 3.5 V, and the second one—to the Co^2+^/Co^3+^ redox at 4.9 V. The structural evolution of LiCo_0.5_Fe_0.5_PO_4_ during cycling was tracked by operando synchrotron XRD. Upon the first charge, the lattice parameter “a” increases; however, the “b, c” decrease. On the other hand, a decreases, and b, c increase during the discharge process. Simultaneously, the electronic and local atomic structure of the material was examined using *operando* synchrotron XAS. The results proved that the Fe^2+^ ions are fully converted to Fe^3+^, however, Co^2^ is only partially oxidized to Co^3+^. The percentage of 3d metals that undergo redox reactions is determined by quantitative analysis of spectroscopic data using PCA. This value is then compared to the number of electrons transported through the cell to confirm the electrochemically active phase. DFT calculations are performed using experimental data as a benchmark. When the theoretical model reproduces the observed values, it may be expanded to include parameters that the experimental setup does not allow for. In this way, we examined the Li_x_CoFePO_4_ performance in the whole range of lithium stoichiometry x = 0 . . . 1. When x is smaller than 0.75 in LixCoFePO_4_, the anionic redox process associated with oxygen atoms in the lattice, which accompanies iron charge shifts and dominates at high voltages, is predicted using DFT and Bader charge analysis.

## Data Availability

The datasets used and/or analyzed during the current study are available from the corresponding author on reasonable request.
